# Climate data for building simulations with urban heat island effects and nature-based solutions

**DOI:** 10.1038/s41597-024-03532-5

**Published:** 2024-07-05

**Authors:** Henry Lu, Abhishek Gaur, Michael Lacasse

**Affiliations:** Construction Research Center, National Research Council Canada, Ottawa, Canada

**Keywords:** Projection and prediction, Climate-change mitigation

## Abstract

As cities face a changing climate, buildings will be subjected to increasing energy demand, heat stress, thermal comfort issues, and decreased service life. Therefore, evaluating building performance under climate change is essential for maintaining sustainable and resilient communities. To better prepare building simulation climate data with urban effects, a computationally efficient approach is used to generate “urbanized” data, where the city’s unique signature is obtained through the dynamic Weather Research and Forecasting model for the Ottawa, Canada region. We demonstrate this process using existing climate data and extend it to prepare projections for scenarios where nature-based solutions, such as increased greenery and albedo, were implemented. The data consists of several 31-year time series of climate variables such as temperature, humidity, wind speed and direction, pressure, cloud cover, and precipitation over different global warming thresholds. Such a dataset allows building practitioners to evaluate building performance under both historical and future climate conditions, as well as to evaluate the impacts of nature-based solutions to mitigate future climate change risks.

## Background & Summary

Cities are at the forefront of climate change impacts, being home to the majority of the global population and built assets, and at the same time, facing challenges such as rising temperatures, increased frequency of extreme weather events, and altered precipitation patterns. The changes in the frequency, magnitude, and duration of the natural hazards as a consequence of a changing climate, can have profound implications for buildings, such as, increased energy demand of buildings, heat stress and thermal comfort issues for building occupants, and decreased service life of building components from exposure to extreme weather events^1,2^. In this context, the evaluation of the performance of buildings under systematically changing climate conditions during their design lives is important for maintaining existing and developing new, sustainable, and resilient communities across the globe.

The evaluation of building performance is commonly conducted by undertaking building simulations, which require climate data files as inputs. The files contain information on a number of climate parameters to which buildings are exposed, such as temperature, humidity, solar radiation, wind speed, and precipitation. The data needs to be provided in high temporal and spatial resolutions to accurately reflect the response of the building to the exterior climate. Typically, climate files reflecting historical patterns are prepared using data recorded at climate gauging stations around the globe. On the other hand, to account for the effects of cliamte change, long-term projected climate data is derived from global climate models (GCMs) which simulate the response of the global climate system under plausible future green house gas pathways^3–5^. Whereas GCMs provide valuable insights into future climate trends^6,7^, their coarse spatial resolution, often ranging in the hundreds of kilometers, limits their utility towards simulating data appropriate for building level impact assessments^8–10^.

Cities have unique climate characteristics because of their complex configurations, especially compared to the rural areas around them^11,12^. For example, the phenomenon of the urban heat island effect (UHI) is well established and understood^13^. It describes the process where urban areas experience elevated temperatures due to human activities and the built environment. Additionally, the aerodynamic roughness of urban surfaces reduces the wind speed through a city, and can alter its prevailing direction^14,15^. While the effects of urbanization on precipitation are still not entirely understood, several factors that affect the precipitation as a result of the built environment have been identified. For example, the building landscape and aerosols from human activities have been shown to alter the precipitation in a city^16,17^. Therefore, when evaluating a building’s response to atmospheric conditions, it is critical to account for these alterations to the local weather pattern. However, these characteristics of urban climate are not well simulated by GCMs or even by regional climate models (RCMs), because the configuration of cities and their morphology, are not well represented. Therefore, to effectively simulate the urban environment, climate simulations are often conducted at convection permitting scales (≤4 km) to achieve the most accurate results^12,18,19^; since they do not rely on convective parameterization schemes and can greatly improve the representation of surface and orographic fields.

To generate GCM projections that are useful for local level impact assessments, it requires that they be spatially downscaled using either statistical and/or dynamical methods^8,20,21^. Statistical downscaling involves developing statistical relationships between large-scale climate variables from GCMs and local-scale variables observed at weather stations. Alternatively, dynamical downscaling, involves using physics-based models to simulate the climate at finer resolutions than traditional GCMs or RCMs. In the past, a large majority of projected climate data intended for use in building simulations have been prepared using statistical downscaling methods^22–24^, where the morphing method is most often used^25^. The morphing method combines historical measured weather data with a climate change signal derived from GCMs to generate future projections for a particular location^26–28^. This method is computationally inexpensive and allows for the quantification of uncertainty in future climate projections, however, statistical methods such as this are prone to deliver unrealistic and physically inconsistent data^29,30^. Gaur and Lacasse (2022) introduced an alternative approach and used long-term climate projections directly simulated by the Canadian Regional Climate Model 4 (CanRCM4) and bias-corrected them using a multivariate quantile delta mapping procedure to prepare building simulation climate data across Canada. This approach allowed them to preserve the inherent variability in the climate system as modelled by CanRCM4, and at the same time correct for biases associated with the simulated variables. However, statistical downscaling methods are not well suited to incorporate complex urban phenomenon, such as UHI effects, in downscaled GCM projections. Ideally, a detailed physics-based model should be used to dynamically downscale the GCM projections.

Consequently, the dynamical downscaling of climate simulations has become a useful tool in understanding the urban climate. For instance, a review of the use of dynamical models to study urban climates found that most studies examined the UHI, followed by local air circulation, surface energy balance, urban planning, air quality, precipitation, thermal comfort, and building energy consumption^31^. One prominently used model is the Weather Research and Forecasting (WRF) model^32^, which is a widely employed numerical convection-permitting mesoscale model often used for short-term weather forecasting and studying UHI^33,34^. To assess the effects of climate change, simulations need to be performed over 30 years to minimize the effects of natural variability of the climate in the analysis. However, this length of simulations with urban effects are rarely performed in experiments due to the high computational costs associated with dynamically downscaling GCMs to kilometer-scale resolutions. While several experiments have performed decadal scale downscaling of GCMs at the continental to city scale^35–37^, the large costs required to perform such simulations makes it difficult to quantify the uncertainty in these results because only a limited number of experiments can be performed. To accurately assess the effects of climate change, one needs to take into consideration multiple climate models, downscaling methods, and initial/boundary conditions into the analysis. As a result, assessing uncertainty in downscaled climate projections, particularly in the building modelling context, remains challenging.

To address this gap, a few novel statistical-dynamical downscaling methods have emerged, leveraging the strengths of both approaches to generate long-term climate projections to explicitly incorporate urban effects. For example, Hoffman *et al*.^38^ studied the evolution of the UHI through a weather pattern classification system. The UHI is obtained by dynamically downscaling to 1-km for representative years, and the average UHI for a particular climate period is statistically reconstructed by comparing the distribution of the weather patterns. Duchêne *et al*.^39^ and Le Roy *et al*.^40^ incorporated the distinct urban signature of a city into long-term RCM data by performing two concurrent dynamic downscaling simulations of the local climate; one considering the urban environment and the other substituting the city scape with natural covers. The urban signature, representing the climate effects of the city, were determined as the difference between these simulations and superimposed on projected RCM data. While these methods demonstrate their capability to capture urban effects, it’s important to note that the data generated from them may not be directly applicable for building simulations; because they are not bias corrected, which is a crucial step for ensuring accuracy in local-scale impact assessments.

Furthermore, in the face of rapidly warming cities due to the combined effects of global warming and urban heat island, some Nature-Based Solutions (NBS) such as increasing greenery and albedo of urban areas, have presented themselves as promising strategies to alleviate the risks of exposure to extreme heat^41–43^. For instance, by increasing the albedo of the surfaces in the urban areas such as the roads, roofs, and walls of buildings, significant cooling benefits can be found^42,44^ and comparable benefits can come from increasing the fraction of urban greenery^45^. Li and Bou-Zeid used the WRF model to downscale re-analysis data to characterize the UHI and subsequently used the results to evaluate the value of NBS such as cool and green roofs^46^. Similarly, Lu *et al*.^47^ analyzed the cooling effectiveness of increasing albedo and vegetation in several Canadian cities. Their findings suggest that the temperature can vary significantly across the same city, and the effectiveness of NBS is highly dependent on the landscape. Therefore, studying the effects of NBS in cities over the long term can provide valuable information for communities to understand the potential benefits different adaptation measures may have on building performance.

In this work, we demonstrate how global climate model data can be modified to prepare building simulation files while incorporating urban effects. The process is used to generate building simulation climate files for the city hall located in Ottawa. Furthermore, the process is extended to prepare projections for cases where two widely used NBS solutions: increased greenery and albedo in urban areas, are implemented in the city. These climate files will allow building practitioners to evaluate the performance of buildings near the city hall of Ottawa under potential future changes in climate taking into account atmospheric interactions with urban morphology and implementation of nature-based solutions.

## Methods

**Fig. 1:**
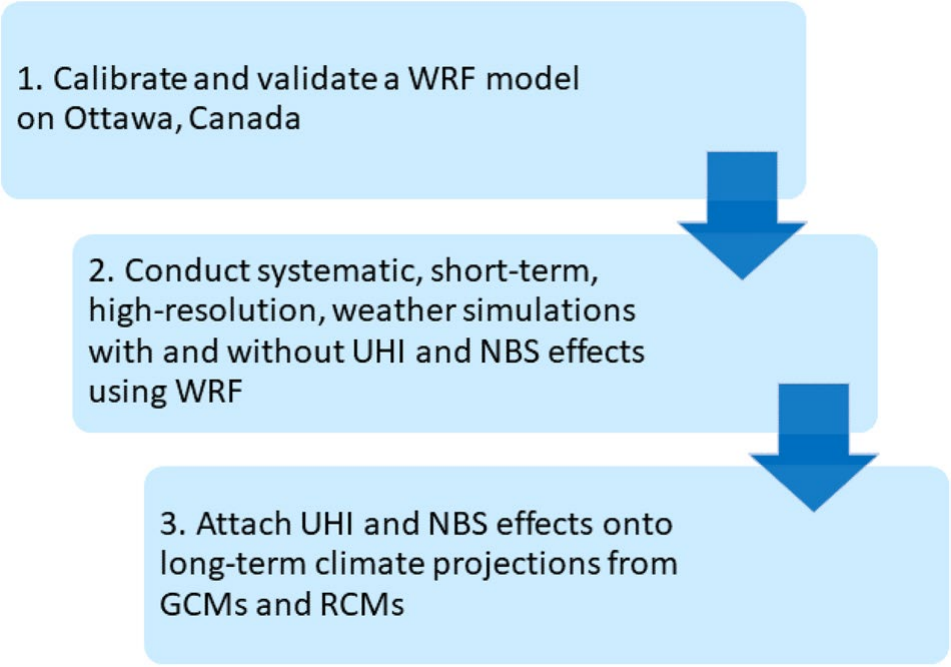
Overview of study design and methodology.

The process to generate long-term urban climate data for the city hall located in Ottawa involves obtaining bias-corrected building simulation climate files for the Ottawa International Airport location as provided by Gaur *et al*. (2022). These are used as a reference dataset devoid of urban or NBS effects, and on which these effects are added by implementing the steps outlined in Fig. 1 and summarised below.

A WRF model at 1 km spatial resolution is configured over regions surrounding the city of Ottawa. The model is used to conduct several experiments with and without urban parameterization and the implementation of nature-based solutions. From an analysis of the differences in these simulations, the urban and NBS effects are isolated, following Duchene *et al*.^39^, and to obtain long-term climate projections, those effects are subsequently acquired at the city hall location and are integrated onto the reference datasets from Gaur *et al*. (2022).

### Study area

**Fig. 2:**
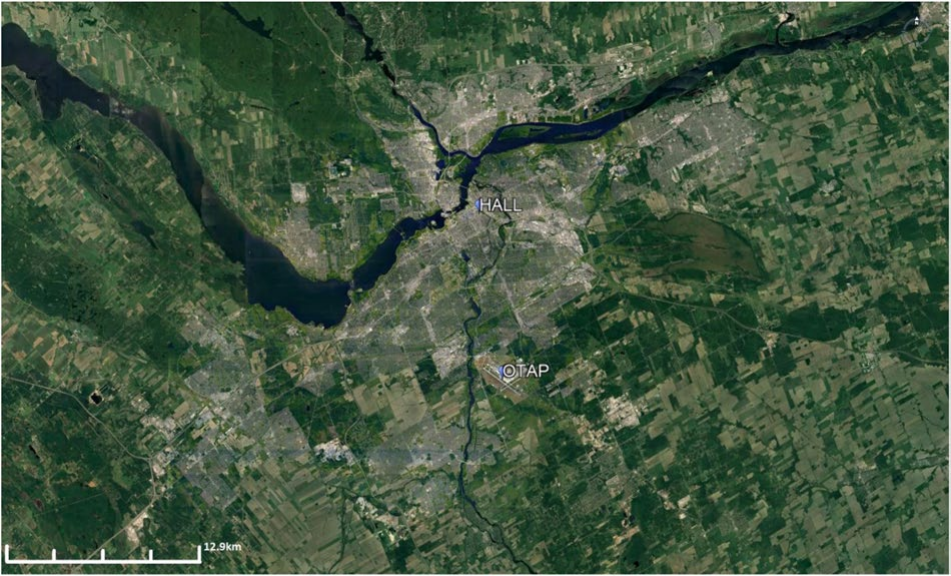
Location of the Ottawa airport weather station (OTAP) and city hall (HALL).

The city of Ottawa is the capital city of Canada with a population of just over 1million people. As shown in Fig. 2, the Ottawa river passes through the northern part of the city, with the city of Gatineau located north of the river and Ottawa to the south. The areas surrounding Ottawa consists mostly of farmland or are otherwise covered by natural vegetation. On the other hand, the developed areas largely consist of open low-rise buildings spread over a large area, and a small dense urban core which is relatively close to city hall. The climate in Ottawa is typically warm and humid in the summer. The city receives nearly 40% of its precipitation during the summer months (May-Aug) at 347.5 mm, and has an average summer temperature of 18.3 °C between 1981–2010. The highest temperature recorded over this period for May, June, July, and August, is 35.0 °C, 36.7 °C, 37.8 °C, and 37.8 °C, respectively.

### Modelling urban and nature-based effects

**Fig. 3:**
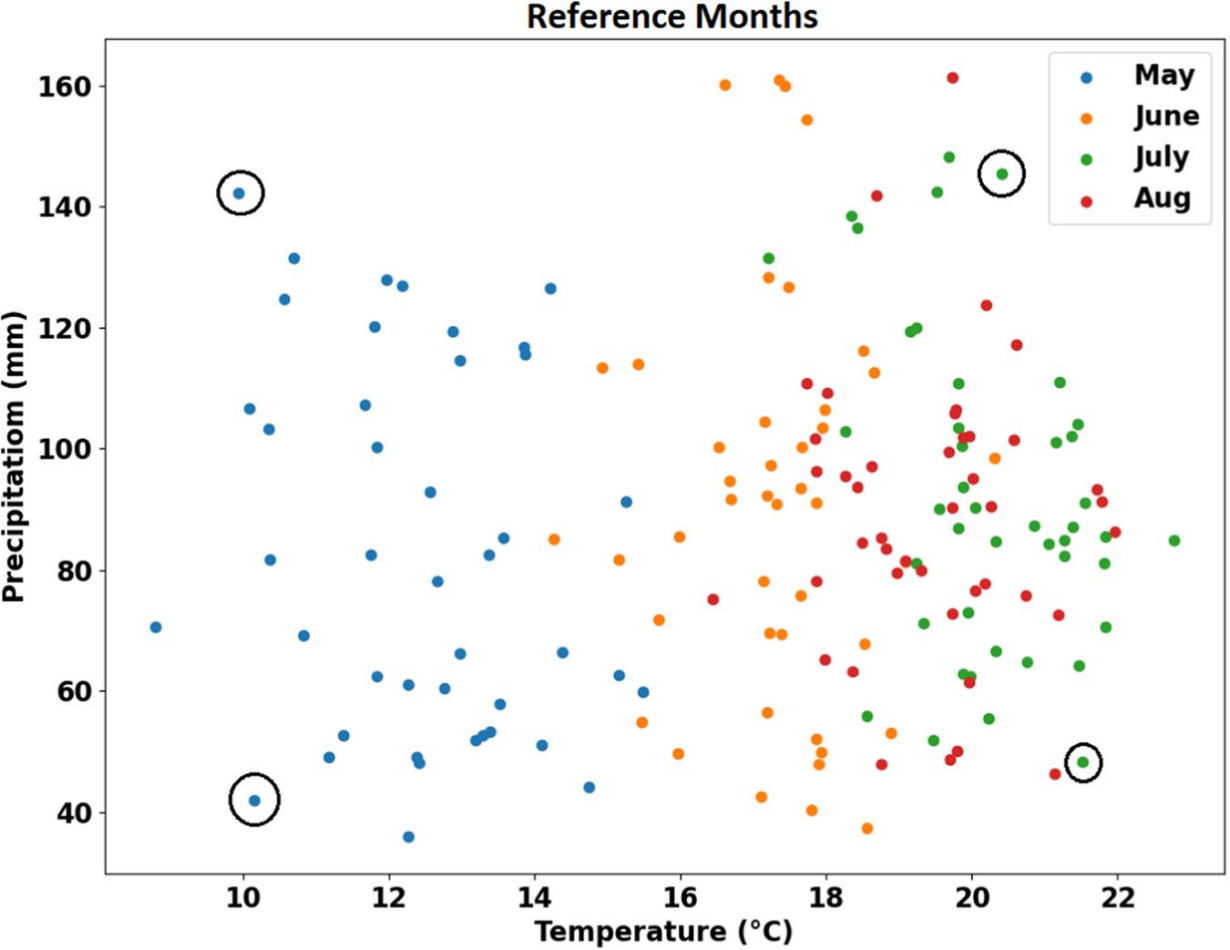
Summer reference months selected (circled) by ranking their monthly average temperature and total precipitation.

The WRF model was used to simulate the climate over the Ottawa region taking into consideration the urban and NBS effects. The North American Regional Re-analysis (NARR)^48^ data was chosen as the initial and boundary condition for the simulations. The urban and NBS effects were modelled for diverse summertime (May-August) climates experienced by this region to prepare a comprehensive database of urban and NBS effects, which were later mapped onto the long-term climate projections. To identify a diverse range of months, all summer months between 1979–2021 were ranked according to their average temperature and total precipitation in the domain. The individual months were given a score based on their rank from highest to lowest temperature, where a small rank indicates warmer temperatures while a large rank represents colder. Additionally, that was added to their rank with respect to the total monthly precipitation, which is calculated in a similar manner. The procedure was performed to find the coldest extreme months as well. In total, four reference summer months were selected for their extreme characteristics, including: the coldest and wettest month (May 1984), the warmest and driest (July 2002), the coldest and driest (May 2005), and the warmest and wettest (July 2008) as illustrated in Fig. 3. Subsequently, WRF simulations of urban and NBS effects were performed over these climatologically diverse months.

**Fig. 4:**
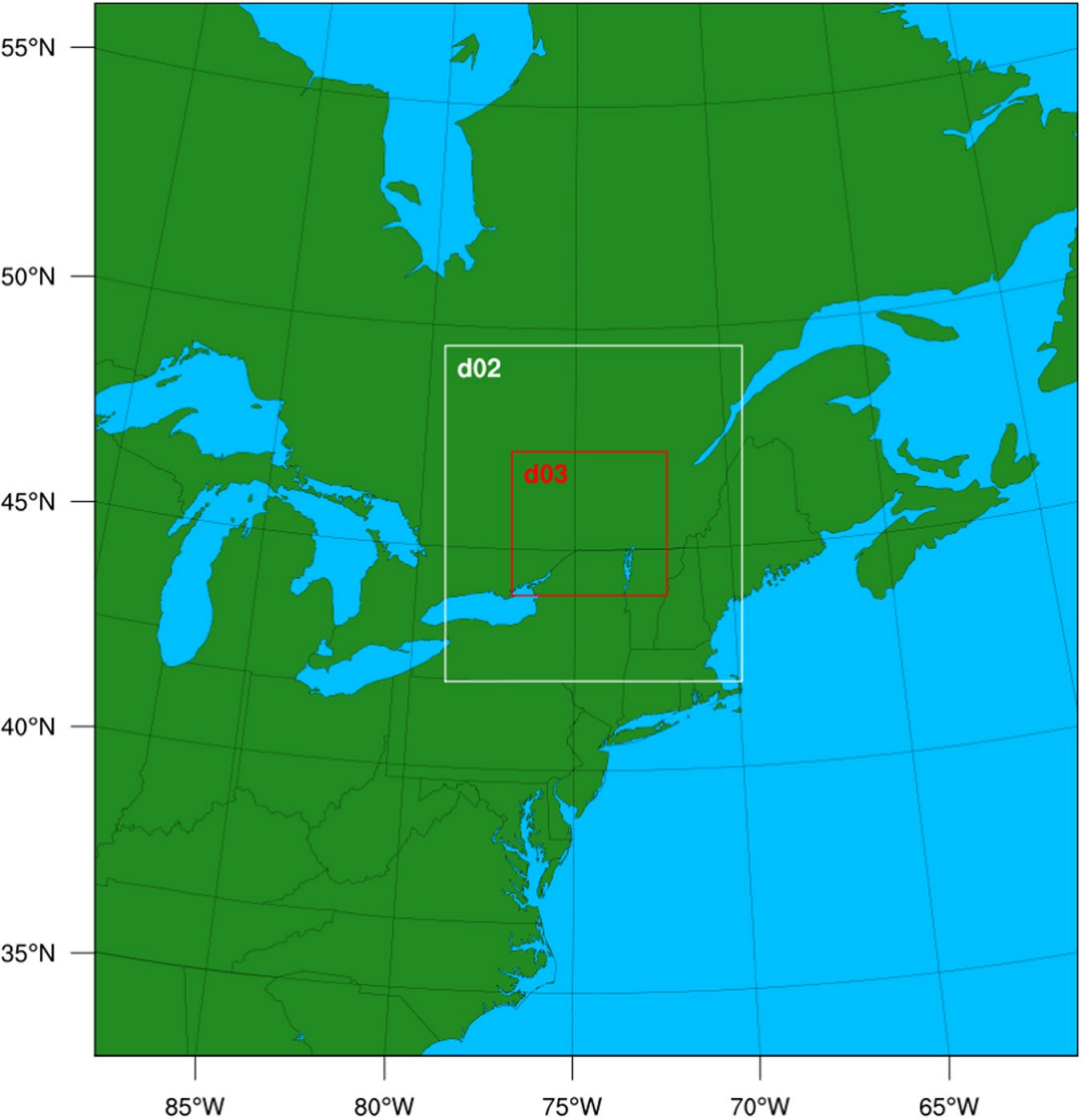
WRF model domains for simulating the city of Ottawa.

To precisely model the urban effects, it was coupled to the multi-layer urban canopy model, Building Effect Parameterization (BEP) and Building Energy Model (BEM)^49^, which simulates the three-dimensional transfer of heat, moisture, and momentum, and allows the urban canopy to directly interact with the planetary boundary layer. While some studies found that multi-layer urban canopy models can lead to poorer model performance due to the difficulty in configuring them^50,51^, it has been shown that this particular model setup, utilizing BEP and BEM, yield the best result for this study area^11,12,52^. The study area illustrated in Fig. 4 consisted of three two-way nested domains with 276 × 296, 250 × 283, and 391 × 364 grid points at a resolution of 9 km, 3 km, and 1 km, respectively. For the purposes of this study, only the 1 km resolution data was analyzed. To select the best physics parameterizations for our study region, several tests were conducted using different combinations of physics options, and it was found that this combination resulted in the best overall accuracy and is well supported by previous experiments^53–55^. This particular WRF model setup, as listed in Table 1, has been extensively validated in previous studies for this specific region over Ottawa, Canada^11,52^, and therefore was not repeated in this study. Overall, a total of 20 different variations of the reference summer months were simulated with WRF.

**Table 1:** WRF physics options used for simulations in Ottawa.

Parameterization	Option
**Microphysics**	WRF Single–Moment 3
**Long Wave Radiation**	RRTM
**Short Wave Radiation**	Dudhia
**Surface Layer**	Eta Similarity
**Land Surface Model**	Unified Noah
**Planetary Boundary Layer**	BouLac
**Cumulus**	Kain–Fritsch (outer domain only)
**Urban**	BEP+BEM

The following scenarios were examined for the baseline reference and NBS effects; these were implemented in WRF by simulating different increasing albedo (ALBD) and greenery (GRN) conditions:

- **Baseline Urban (UP)**: The baseline urban scenario was performed as the control experiment where the model was intended to represent real conditions. To that end, the most appropriate inputs were used, such as a 100 m local climate zone map^56,57^ to represent detailed land use and cover in the city. In addition to an active urban canopy model, the most realistic urban parameters were used.
- **Non-Urban (noUP)**: The urban canopy model was deactivated and represented a scenario in which the city was replaced with natural vegetation. From these differences in the baseline urban and non-urban scenarios, the urban signature was derived.

- **Scenario 1 (ALBD)**: In this scenario, the albedo of roofs was increased to 0.80, whereas the roads and walls of buildings were increased to 0.40. A choice was made to keep the albedo of roads and walls at a lower level (0.4) as compared to roofs in all scenarios due to the potential adverse effects that highly reflective materials could have on pedestrians and drivers when implemented on the ground and wall surfaces^58^.

- **Scenario 2 (GRN)**: In this scenario, the urban vegetation fraction was increased to cover 80% of the existing urban areas. This is modelled by replacing the existing built surfaces with vegetation.
- **Scenario 3 (COMB)**: In this scenario, a combination of the previous two scenarios was used, i.e., both albedo and greenery were modified simultaneously as discussed above.

Additionally, we have simulated less extreme variations of these NBS scenarios, where the values were set to 0.40 or 40% for albedo and greenery, respectively; as well as a combined case. Although these results were not discussed in the following sections, the data is made available as referenced in the data records.

### Isolating signatures

The simulations with and without urban and NBS effects were used to calculate the urban signature or nature-based signature as defined in Eq. (1), for variable *i*, at location *r* and *s*, and time *t*. For each day in the database (i.e. 4 reference months), a signature was acquired by calculating the difference between the baseline urban scenario (UP), denoted *X*^*UP*^ (*d)*, and one of the modified scenarios (i.e. noUP, ALBD, GRN, or COMB), *X*(*d)*. We define the urban signature as the difference between the UP and noUP cases, while the nature-based signature is the difference between the UP and ALBD, GRN, or COMB scenarios. The signatures were acquired for each climate variable, including: global horizontal irradiance, rainfall, relative humidity, wind speed, wind direction, total cloud cover, temperature, atmospheric pressure.

1 $${{Signature}}_{i,r,t}(d)={{X}_{i,r,t}}^{{UP}}(d)-{X}_{i,s,t}(d)$$

### Integrating signatures onto climate projections

**Fig. 5:**
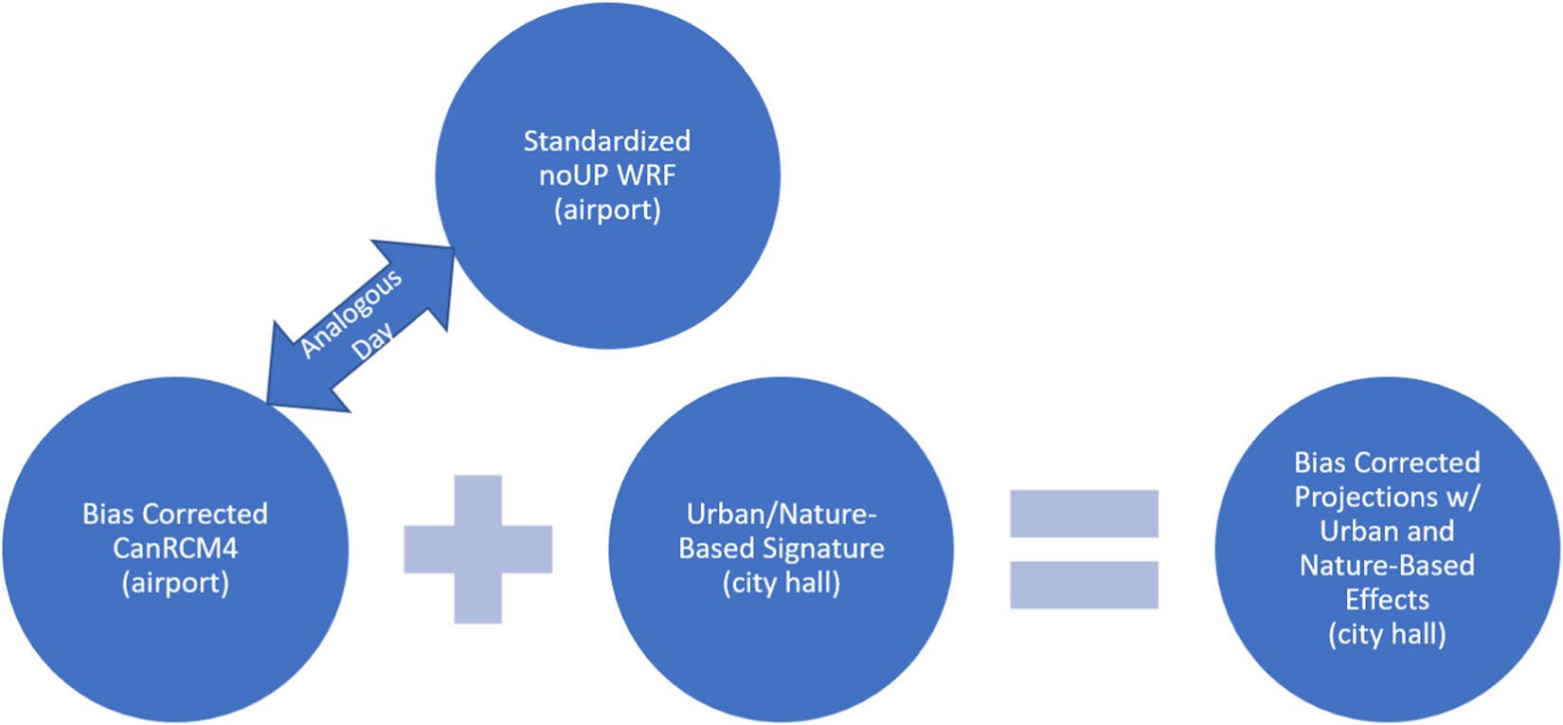
Process to integrate urban and nature-based effects onto long-term climate projections.

The integration of urban and NBS effects onto the RCM data was conducted by finding an analogous day between the RCM and noUP dataset. The analogous day was calculated by first standardizing each variable for each day. Then the analogous day (*d*_*min*_) was found by minimizing the cost function described in Eq. (2), where $\bar{X}$ represents the standardized data, for each climate variable *i*, at all spatial points *r*, and all times of day *t*. In this case, the spatial points considered were those within the boundaries of the inner-most domain of the WRF model. Lastly, the RCM data was calibrated by adding the signature acquired in Eq. (1), following Eq. (3), where ${{X}_{r}}^{{RCM}}$ is the regional climate model projection bias corrected with respect to a climate gauging station at *r* (commonly available at airports), and *Signature*_*s*_ is the signature dervied at an arbitrary location *s* in the city (in this case, Ottawa city hall). Therefore, the resultant data is an urbanized version of bias-corrected climate projections, originally situated for the airport, which can now be used as inputs to building simulations near Ottawa city hall (Fig. 5).

2 $$C(d)=\sum _{i}\sum _{r}\sum _{t}{(\overline{{{X}_{i,r,t}}^{{RCM}}({d}_{{RCM}})}-\overline{{{X}_{i,r,t}}^{{noUP}}({d}_{{noUP}})})}^{2}$$

3 $${Y}_{i,r,t}={{X}_{i,r,t}}^{{RCM}}({d}_{{RCM}})+{{Signature}}_{i,s,t}({d}_{mi\mathrm{in}})$$

### Regional climate model data

The RCM data that was used in this step comes from the Canadian Regional Climate Model 4 (CanRCM4) Large Ensemble data^59^, which consists of 15 realizations with slightly perturbed initial conditions, and was run continuously from 1950–2100 under RCP8.5 with an hourly time step. The CanRCM4 ensemble was divided into several 31-year ranges which correspond to different levels of global warming thresholds compared to the historical period^60^, as listed in Table 2. Furthermore, the data was bias-corrected with reference to a local climate gauging station located at the Ottawa International Airport (OTAP). The multivariate bias correction with N-dimensional probability density function transform (MBCn) was chosen as it is able to correct the marginal distribution of variables and the dependence structure between them^61^. Studies have shown the importance of considering the internal variability of climate when bias-correcting an ensemble of climate model data^62,63^. Therefore, data was generated following the proposed methodology for all 15 runs of the bias corrected RCM to allow users to take into account the uncertainty of climate change. However, the following results are discussed as an average of the whole ensemble, unless otherwise specified.

**Table 2:** Historical and future global warming thresholds and their corresponding time periods.

Global Warming Scenario	Time Period
**Historical**	1991–2021
**Global Warming 0.5 °C**	2003–2033
**Global Warming 1.0 °C**	2014–2044
**Global Warming 1.5 °C**	2024–2054
**Global Warming 2.0 °C**	2034–2064
**Global Warming 2.5 °C**	2042–2072
**Global Warming 3.0 °C**	2051–2081
**Global Warming 3.5 °C**	2064–2094

## Data Records

The full dataset is publicly available at: https://zenodo.org/records/11243998^64^. This includes all the time periods listed in Table 2 and hourly variables from Table 3, as well as several other NBS scenarios which were not discussed. The data are stored in large CSV files, where the rows consists of all 15 realizations of the CanRCM4 ensemble and the variables make up the columns. For example, each 31-year period is repeated 15 times, once for each of the RCM realizations. Therefore, there are 4,073,400 (15 × 31 × 8760) rows in each file. The column names and detailed description of what each represents is shown in Table 4.

**Table 3:** Generated climate variables for building simulations.

Climate Variable	Units
**Global Horizontal Irradiance**	kJ/m^2^
**Rainfall**	mm
**Relative Humidity**	%
**Wind Speed**	m/s
**Wind Direction**	Degrees clockwise from North
**Total Cloud Cover**	%
**Temperature**	°C
**Atmospheric Pressure**	Pa
**Snow Depth**	cm

**Table 4:** Variable name and description of the climate data in CSV files.

Variable	Description
RUN	Run number (R1-R15) of Canadian Regional Climate Model, CanRCM4 large ensemble associated with the selected reference year data
YEAR	Year associated with the record
MONTH	Month associated with the record
DAY	Day of the month associated with the record
HOUR	Hour associated with the record
YDAY	Day of the year associated with the record
DRI_kJPerM2	Direct horizontal irradiance in kJ/m2 (total from previous HOUR to the HOUR indicated)
DHI_kJperM2	Diffused horizontal irradiance in kJ/m2 (total from previous HOUR to the HOUR indicated)
DNI_kJperM2	Direct normal irradiance in kJ/m2 (total from previous HOUR to the *HOUR* indicated)
GHI_kJperM2	Global horizontal irradiance in kJ/m2 (total from previous HOUR to the HOUR indicated)
TCC_Percent	Instantaneous total cloud cover at the HOUR in % (range: 0–100)
RAIN_Mm	Total rainfall in mm (total from previous HOUR to the HOUR indicated)
WDIR_ClockwiseDegFromNorth	Instantaneous wind direction at the HOUR in degrees (measured clockwise from the North)
WSP_MPerSec	Instantaneous wind speed at the HOUR in meters/sec
RHUM_Percent	Instantaneous relative humidity at the HOUR in %
TEMP_K	Instantaneous temperature at the HOUR in Kelvin
ATMPR_Pa	Instantaneous atmospheric pressure at the HOUR in Pascal
SnowC_Yes1No0	Instantaneous snow-cover at the HOUR (1 - snow; 0 - no snow)
SNWD_Cm	Instantaneous snow depth at the HOUR in cm

## Technical Validation

### Assessing climate data with urban effects

To evaluate the suitability of the methodology, a split-sample approach was used. WRF simulations with and without urban and NBS effects were conducted over the four diverse reference months selected for this region. In the split-sample approach, the entire sample of WRF simulations of the four selected months is randomly divided into two equal halves: a training dataset and a validation dataset. The training dataset is used to isolate the urban and nature-based solution effects from the WRF experiments. These effects are then integrated into the non-urban WRF results to predict a new dataset that emulates WRF simulations with urban and NBS effects. This predicted dataset is compared to results directly obtained from WRF simulations with urban and NBS effects over the validation time-period to evaluate the performance of the new data.

**Fig. 6:**
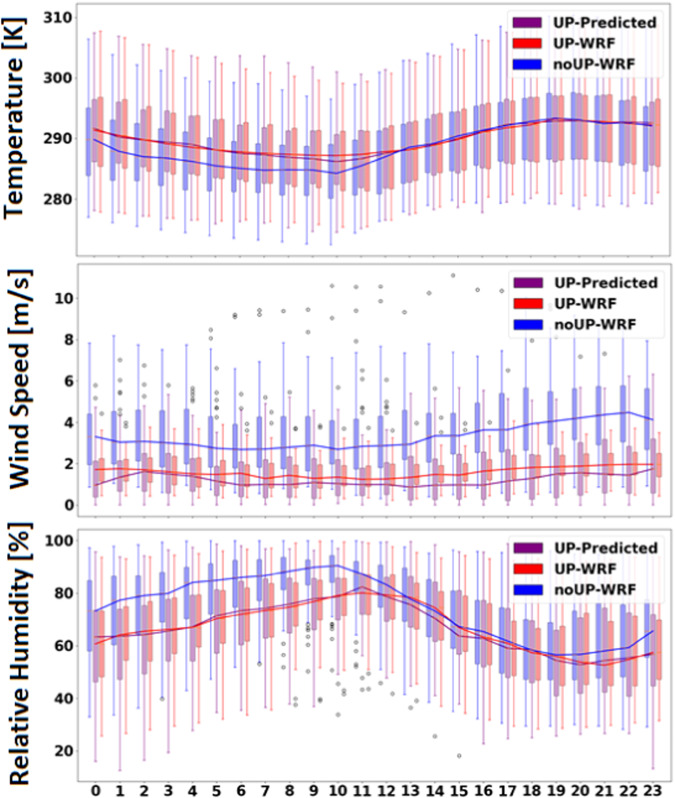
Diurnal cycle of the calibrated temperature, wind speed, and relative humidity compared with the WRF simulations with and without urban parameterizations. Boxplots represent the spread at each hour (considering all days) and the solid line shows the mean. The analysis is only conducted for WRF grids in the domain considered to be urban.

The diurnal variations of the three climate variables obtained for the validation period from noUP-WRF, UP-WRF, and UP-Predicted cases are shown in Fig. 6. We find that the UP-Predicted data is better aligned with UP-WRF than the noUP-WRF profile. For example, the temperature is significantly improved during the morning hours, where the difference between noUP-WRF and UP-WRF is greatest. Meanwhile, the differences during the night between the UP-Predicted, UP-WRF, and noUP-WRF data are more subtle. A similar pattern emerges from the comparison of relative humidity, where UP-Predicted and UP-WRF humidity are well aligned in the morning. Lastly the wind speed in the UP-WRF and UP-Predicted case are expectantly lower than the noUP-WRF case, consistently by 2 m/s throughout the day. The improvements in temperature, relative humidity, and wind speed can be attributed to the approach’s ability to capture the localized effects of the urban canopy. For instance, in urban areas, buildings and pavement absorb and retain heat, leading to higher temperatures compared to non-urban areas. Therefore, by incorporating urban effects back into the noUP-WRF data, the model can better replicate the physical urban environment. Similarly, urban structures can alter wind patterns by creating drag or re-directing airflow. The model predicts lower wind speeds in areas with increased building density, leading to better alignment with wind speeds in UP-WRF simulations.

The mean bias (MBE) and root mean square error (RMSE) is calculated between the UP-Predicted and UP-WRF data, as shown in Table 5. The calculation includes data for the entire Ottawa region that is classified with urban land cover, and ignoring natural land cover types in the domain. This subsetting of the WRF model domain eliminates the negligible effects that are present in the model grids that have natural vegetation coverage, and which may negatively skew the results. The results shows that there is an extremely small bias between these two datasets, with a slight positive bias (0.02 K) in near-surface air temperature, and slight negative bias in wind speed and relative humidity at −0.03 m/s and −0.4%, respectively.

**Table 5:** Mean bias and root mean square error of the calibrated data compared to the WRF modelled urban data.

	TEMP [K]	WSP [m/s]	RH [%]
**MBE**	0.019	−0.033	−0.38
**RMSE**	1.17	1.12	7.72

### Climate projections

The results for the historical period show that the methodology used in this study can reliably integrate and mimic urban and NBS effects onto a “non-urbanized” climate dataset. The methodology is expanded to prepare projected building simulation climate files for a highly urbanized location in Ottawa, near the city hall. As discussed earlier, an existing set of building simulation climate files^60^ was used as the baseline RCM data, to which urban and NBS effects will be added. To integrate urban and NBS effects of the city hall location onto the airport location, their signatures are calculated as the difference between the WRF simulated data at the airport versus the city hall. Thus generating urbanized and bias corrected climate data at the city hall which can be used for building simulations. Finally, the results will focus on the historical (1991–2021) and 3.5 °C of global warming (2064–2094) scenarios to be concise and to clearly validate the most extreme climate change scenario. However, data will also be accessible for other global warming periods.

**Fig. 7:**
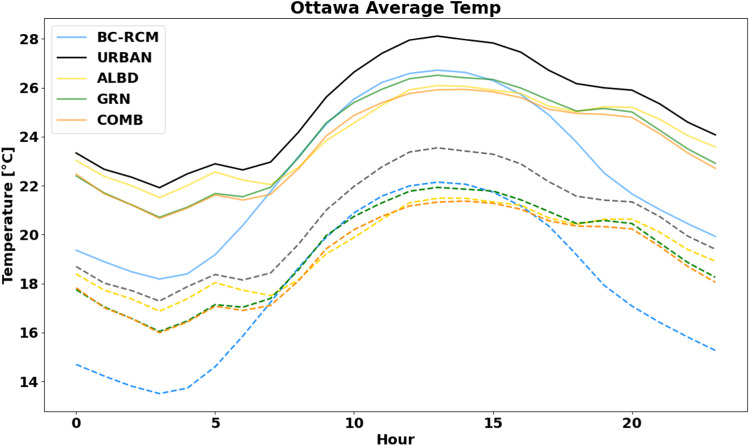
Summertime average of the diurnal pattern for the urban and nature-based cases compared to the reference bias-corrected CanRCM4 (BC-RCM) data during the historical (dashed line) and future (solid line) time period under 3.5 °C of global warming.

Figure 7 is a result of combining the urban and nature-based signatures with the bias-corrected CanRCM4 (BC-RCM) for data under the historical and 3.5 °C global warming scenarios. The diurnal profile is calculated as an average of 15 CanRCM4 realizations and across a 31-year period. As expected, the daytime temperature is somewhat greater (~2 °C) when interactions between the urban canopy and atmosphere are considered for the city (URBAN)^65–67^. However, in the presence of NBS such as increasing albedo (ALBD) or greenery (GRN), we observe a marginal decrease in the daytime air temperatures compared to the BC-RCM and especially the URBAN case. Nighttime temperatures are much more elevated due to the UHI effect, which may be as high as 5 °C whereas, no mitigation solutions were in place. The impacts of increasing albedo at night is marginal, where the muted effect is likely a result of the reduced heat absorbed by construction materials during the day. Alternatively, increasing the greenery yields a consistent cooling effect throughout the day and night. Lastly, the combined case (COMB) gets the benefits of increased albedo during the day and moderating effects of more vegetation at night. In the early evening hours (18:00–20:00), we observed somewhat elevated temperatures even though it becomes cooler at night.

Under 3.5 °C of global warming, the diurnal profile closely resembles the relative difference between those found in the historical scenario. As the scenario implies, the BC-RCM data is observed to be 3–4 °C greater during this period than in the historical. While the analogous days selected for the historical and projected period are different, the resultant urban effects exhibit similar diurnal patterns and comparable cooling effects. It is of interest to note that the average temperature of the historical URBAN dataset is nearly as warm as the future BC-RCM data at night. This implies that the historical nighttime UHI effect is similar to the end of century global warming magnitude under RCP8.5, illustrating the severe underestimation of nighttime air temperature as would typically be encountered from the use of this climate data for building simulations.

**Fig. 8:**
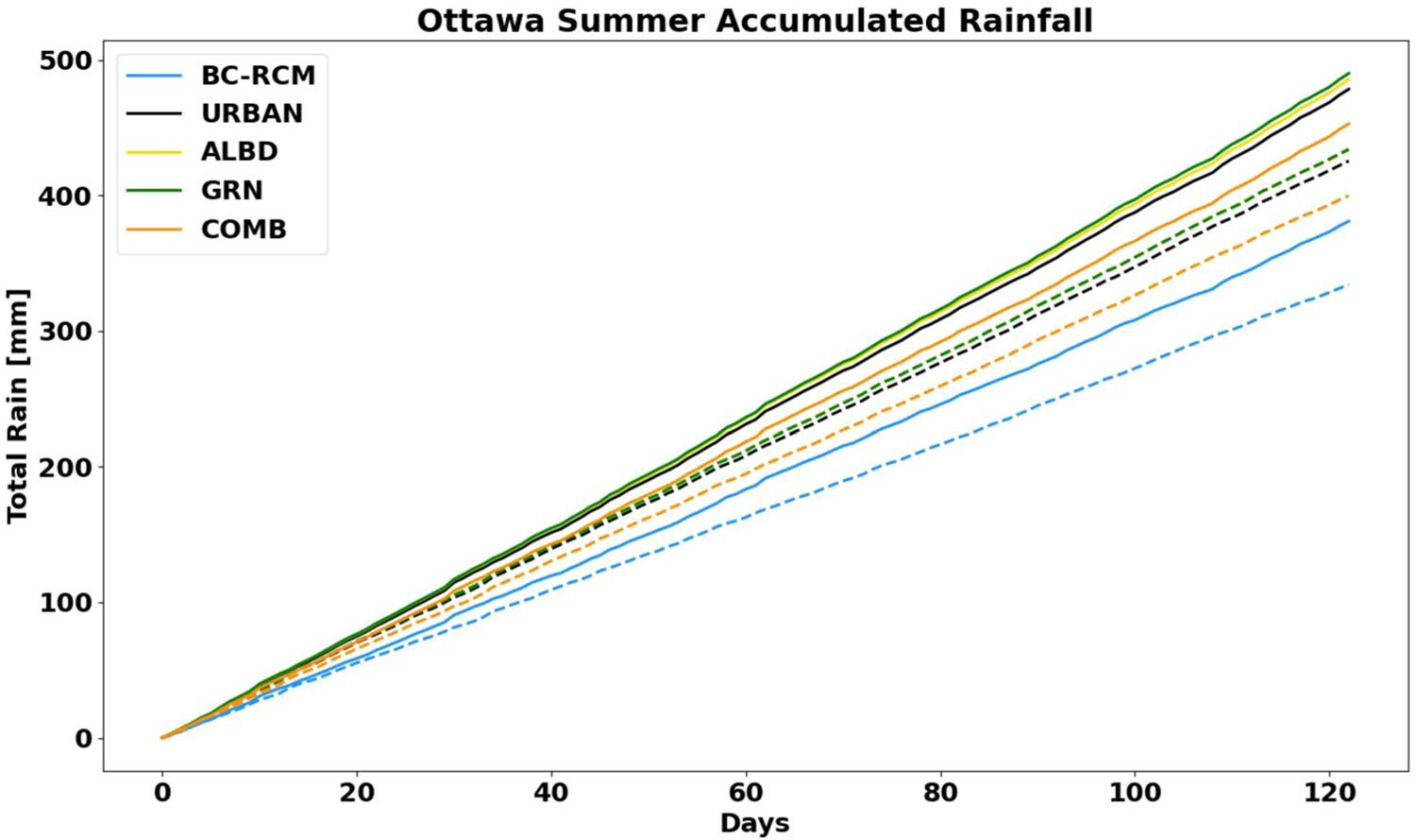
The average summer accumulated rainfall over for the historical (dashed line) and global warming (solid line) period.

Similar to temperature, we find that climate change will lead to an increase in the total accumulated rainfall during the summer, as illustrated in Fig. 8. Consistent with many past findings that suggest climate change will lead to more precipitation^68–70^, in Ottawa, this will lead to an overall increase of 50 mm of accumulated rainfall over the summer time. Examining the different cases, we also find that the URBAN, ALBD, GRN, and COMB data exhibit significantly more rainfall than the BC-RCM data. As a result of the coarse spatial resolution (~50 km) of the raw CanRCM4 data, the CanRCM4 model relies on the parameterization of convection which is a known source of uncertainty in RCMs^12,18,19^. On the other hand, the primary improvements brought by convection permitting climate models is found in the intensity of extreme precipitation events which are a result of the explicit handling of deep convection and the integration of more realistic model dynamics. In fact, when combining the BC-RCM with the downscaling approach outlined in our methodology, which includes the urban effects in WRF simulations at convection permitting scales, the URBAN data estimates significantly more rainfall accumulated throughout the summer in Ottawa. For example, there is nearly a 100 mm difference between the BC-RCM and URBAN case, for both the historical and projected period.

**Fig. 9:**
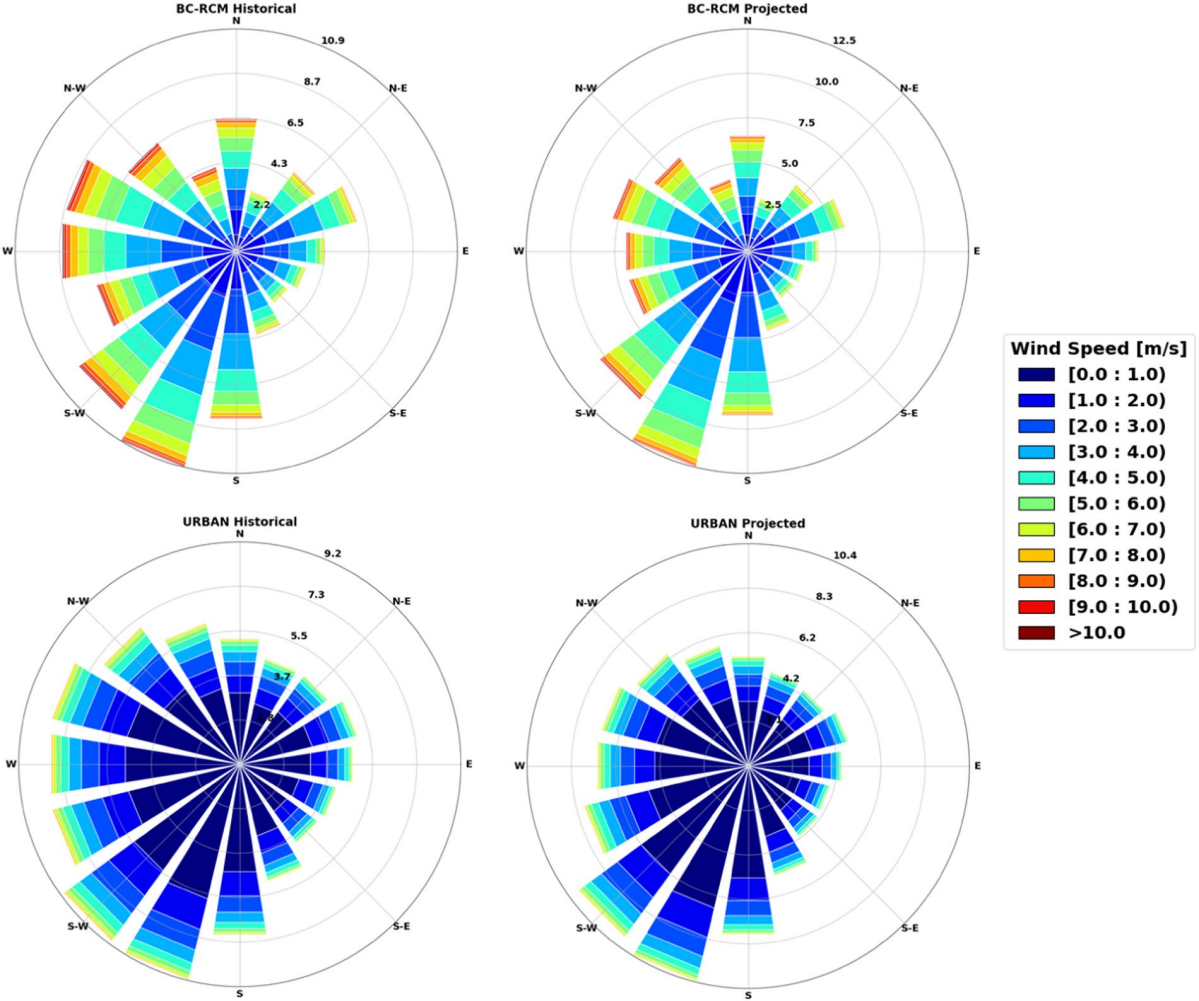
Wind rose for the historical and projected period in Ottawa for the bias-corrected CanRCM and URBAN timeseries.

Figure 9 shows the distribution of wind speed and wind direction for all of the different cases under the historical and climate change scenario. In these figures, we observe extreme differences in the wind speed between the BC-RCM and URBAN data. The wind speeds are often greater than 5 m/s in the BC-RCM case, with a smaller frequency of speeds less than 5 m/s. However, by correcting for the urban effects in Ottawa, we find significant reductions in the overall windspeed, where it is most often between 0–2 m/s. One reason for the reduced windspeed when considering urban effects is that the urban boundary layer poses a larger aerodynamic roughness length than the surrounding natural environments. This roughness results in greater frictional resistance to airflow, slowing down wind speeds compared to surrounding natural environments^71–73^. This comparison is also applicable with the BC-RCM case since the CanRCM4, of which it is based, does not have urban parameterizations, and therefore the urban area acts as if it is covered by natural vegetation. This phenomenon is further exacerbated by the compact layout of urban infrastructure, which can cause wind to be channeled and deflected around buildings, leading to lower wind speeds in urban canyons and streets. The discrepancy highlights the importance to accurately incorporate urban features and their effects on airflow dynamics in climate data to improve the representation of wind patterns. Additionally, comparing the prevailing wind direction between these two cases, we find that the BC-RCM data shows a common westerly wind pattern, but the dominant winds come from the south-west and less frequently from the east. On the other hand, URBAN data exhibits a more uniform distribution of the wind direction, with more wind coming from the north and east. However, the prevailing winds in this case still come from the south-west.

## Usage Notes

While we focused on the city of Ottawa, these general ideas can be applied to any period and location to generate a continuous long-term series of climate data for any city. Additionally, whereas more emphasis was placed on the temperature, the process is applicable for many other climate variables such as precipitation, wind direction, cloud cover, and pressure, as listed in Table 3. By combining the bias corrected RCM data with the urban or nature-based signature, we are essentially overlaying the urban effects experienced at the urban center with reliable climate data at the airport. This transposing between the two locations helped to generate robust urban climate data at Ottawa’s city hall which will be useful for climate change impact analyses on buildings.

The dataset generated through our study provides a comprehensive resource for evaluating building performance and urban climate resilience in the context of a changing climate. The dataset includes hourly climate data for Ottawa, Canada, spanning the period from 1991 to 2094. It encompasses various global warming scenarios (0.5–3.5 °C), urban heat mitigation strategies (changes in albedo and greenery), and 15 ensemble members. The data files are organized by scenario and time period for ease of use. It is tailored for use in building hygrothermal and energy modeling software. We expect practioners to perform some pre-processing of the data files before inputting into a building model, for example, to find a typical/extreme warm year out of the 31-year 15-member ensemble. We recommend the data be processed through a simple Python or R script.

## Data Availability

The custom Python and R scripts used to extract CanRCM4 data, calculate the signatures, and generate projected UHI and NBS climate data for building simulations can be found on Github at: https://github.com/henrylu2/Climate-projections-to-support-building-adaptation.
